# A 74-year-old woman with a 1-month history of itching and skin rash

**DOI:** 10.5144/0256-4947.2009.234

**Published:** 2009

**Authors:** Sujoy Ghosh, Arjun K. Ghosh, Andrew Collier

**Affiliations:** From The Ayr Hospital General Medicine, Ayr, United Kingdom

A 74-year-old woman presented with a 1-month history of itching followed by a rash. The bullous lesions were large and wide-spread (including lesions on the arm, legs and torso, covering almost 20% of the body surface) (Figure 1). Many lesions were de-roofed and there was evidence of underlying inflammation and the presence of marginal vesiculation. She was hypothyroid (taking levothyroxine) and hypertensive (taking bendroflumethiazide) and had a history of a cerebrovascular accident for which she was taking aspirin. She had a fracture of the shaft of the left femur 3 weeks prior to presentation. She was pale with hemoglobin of 8.1 g/dL with normal renal and hepatic function.

**Figure 1:**
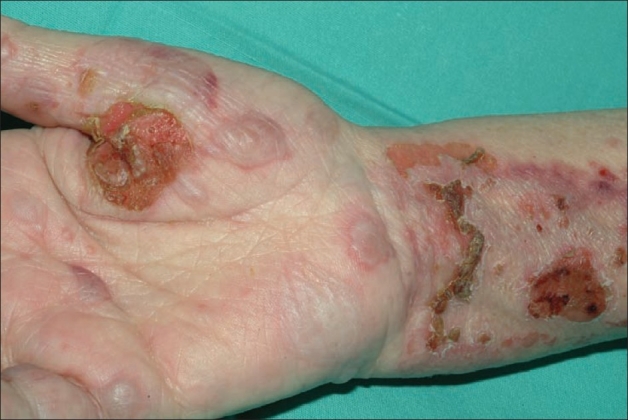
Multiple lesions in different stages of evolution.

What is your diagnosis?

FOR THE ANSWER, VISIT:

http://www.saudiannals.net

## Diagnosis: Bullous Pemphigoid

**Figure 2:**
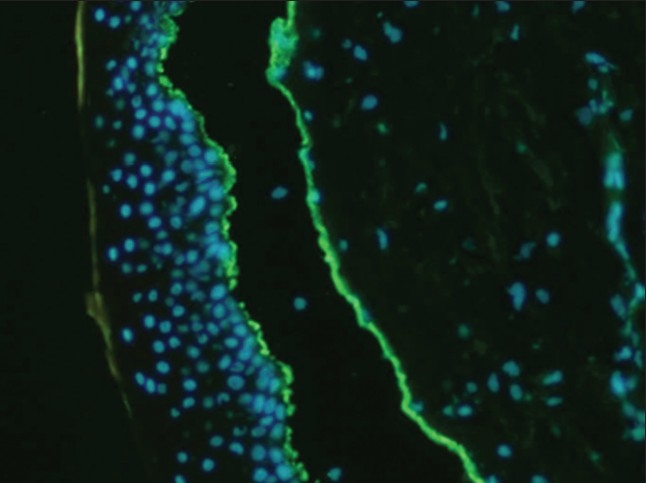
Immunofluorescence study showing IgG positivity at the dermo-epidermal junction

**Figure 3:**
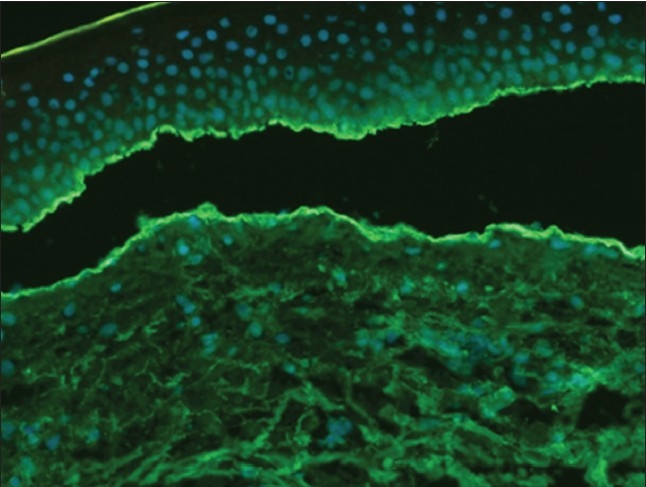
Immunofluorescence study showing C3 positivity at the dermo-epidermal junction

This patient presented with bullous lesions that were large, blistering and itchy, and that sub-sequently underwent excoriation. There was evidence of underlying inflammation and a skin biopsy showed subepidermal blister with adjacent excoriation. There was some evidence of inflammatory cell infiltrate in the floor and a few eosinophils. Immunofluorescence studies showed strong IgG and C3 positivity at the dermo-epidermal junction (Figure 2, 3), which was diagnostic of bullous pemphigoid. She was treated with oral steroids (prednisolone, initially 70 mg/day) and topical mupirocin and doing well after one year.

## DISCUSSION

Bullous pemphigoid (BP) is a common autoimmune blistering disease with an annual incidence of 6.1 to 7 per million.^1^ It is almost twice as common in men as compared to women.^2^ The disease is usually self-limitiing, but may last from days to months and rarely even up to ten years.^3^ The co-existence of other autoimmune disease is not uncommon.^4^ The clinical hallmark of BP is the presence of widespread tense bullae, which may arise from normal (non-inflamed bullae) or erythematous (inflammatory bullae). They can occur anywhere, but there is a predilection for the groin, lower abdomen and the flexural surface of limbs.^5^

Many clinical variants have been described based on positive immunofluorescence studies.^6^ Several drugs have been implicated in precipitating a clinical heterogeneous group of bullous disorders similar to BP.^7^ Other differential diagnoses include IgA bullous dermatosis, bullous systemic lupus erythematosus, epidermolysis bullous acquista and pemphigoid. Clinical, histological and immunopathological techniques are used to confirm the diagnosis, including direct immunoflourescence for IgG, IgM, IgA and C3. The biopsy site should preferably be from the upper body and perilesional. Direct immune electron microscopy is the gold standard for antibody localization within the basement membrane zone.^8^

BP is generally regarded as a benign, self-limited disease. Exacerbations and remissions are common and tend to be milder than the initial episode.^9^ The mortality rate averages approximately 27%.^10^ Most patients affected by BP are elderly, having multiple co-morbidities and infection, and co-morbidities usually account for mortality. Localized disease is generally self-limited and responds to potent topical corticosteroids.^11^ For more widespread disease systemic steroids have been used, both oral and high-dose, pulsed intravenous corticosteroids.^12^ Several studies suggest that antibacterial agents like erythromycin, minocycline or dapsone may control disease activity. In addition, other immunosuppressives like azathioprine, methotrexate, leflunomide or mycophenolate mofetil may have some role in management, especially as an adjuvant to corticosteroid therapy. In a small number of cases intravenous immunoglobulins have been used with variable success.^13^
